# Supplementary material for: Neutral genomic signatures of host-parasite coevolution
Source: BMC Evol Biol. 2019 Dec 19;19:230. doi: 10.1186/s12862-019-1556-3 (PMC6924072; doi:10.1186/s12862-019-1556-3)
Supplement: Supplementary file 1 — Additional file 1 In the Supplementary Information, we provide additional methodological details as well as analytical and computational results. [file 12862_2019_1556_MOESM1_ESM.pdf]

Supplementary Information

Neutral genomic signatures of host-parasite coevolution

Daniel Živković, Sona John, Mélissa Verin, Wolfgang Stephan, Aurélien Tellier

.

SI 1. The host effective population size over time

From (1) and (2) we immediately obtain

$$\frac{dN^{\mathrm{W}}}{dt} = \sum_{i=1}^{A} H_i \left[b_i(1-c_{H_i}) - d_i\right] + \sum_{i=1}^{A} b_i(1-c_{H_i}) \sum_{j=1}^{A}(1-s_{ij})I_{ij} - \sum_{i=1}^{A}\sum_{j=1}^{A}(d_i+\delta_{ij})I_{ij}.$$

Assuming that $s_{ij} = s_i$, $\delta_{ij} = \delta_i$ (*i.e.* both parameters being independent of the parasite genotype) and setting dN^{W}/dt to zero, we have

$$\sum_{i=1}^{A} H_i \left[b_i(1-c_{H_i}) - d_i\right] + \sum_{i=1}^{A} \left[b_i(1-c_{H_i})(1-s_i) - (d_i+\delta_i)\right] \sum_{j=1}^{A} I_{ij} = 0.$$

It particularly follows that $s_i = \delta_i = 0$ requires $d_i = b_i(1-c_{H_i})$ to have a constant population size in the host for arbitrary choices of H_i and I_{ij}.

SI 2. Basic reproduction ratios

From (2), we obtain

$$\frac{dP_j}{dt} \quad = \quad -\sum_{i=1}^{A} I_{ij}(d_i+\delta_{ij}) + H_i \left[\alpha_{ij}\beta_{ij}(1-c_{P_j}) \sum_{k=1}^{A} I_{kj}\right].$$

Assuming that $d_i = d$ and $\delta_{ij} = \delta_j$ (*i.e.* both parameters being independent of the host genotype), the former equation simplifies to

$$\frac{dP_j}{dt} \quad = \quad P_j \left[-(d+\delta_j) + \sum_{i=1}^{A} H_i\alpha_{ij}\beta_{ij}(1-c_{P_j})\right].$$

The reproduction ratios of the parasite genotypes are given by

$$R_{0,j} = \sum_{i=1}^{A} H_i\alpha_{ij}\beta_{ij}(1-c_{P_j})/(d+\delta_j).$$

If all $R_{0,j} < 1$, the parasite genotypes are eliminated since they kill more hosts than they infect new healthy ones. When one of these ratios is greater than one, the corresponding parasite genotype is maintained in the population.

SI 3. Fixed points of the dynamical system

We calculated the fixed points by setting (1) and (2) to zero. Note that the solutions were first obtained in terms of the numbers of healthy and infected hosts before being added up to obtain the fixed points of hosts and parasites assuming one parasite per host. The results for the two-allele case are summarized below. For the MA and iGFG model the results for more than two alleles correspond to those of two alleles, whereas results for the iMA and the GFG model can only be obtained for special cases when $A = 3$, but the solutions are not enlightening.

$$\text{Define} \quad u_i = \frac{(b_i(1-c_{H_i})s_{ii} + \delta_{ii})(\delta_{ii} + d_i)}{\beta_{ii}(-b_i(1-c_{H_i})(1-s_{ii}) + \delta_{ii} + d_i)} \quad \text{and} \quad v_i = \frac{(b_i(1-c_{H_i}) - d_i)(\delta_{ii} + d_i)}{\beta(-b_i(1-c_{H_i})(1-s_{ii}) + \delta_{ii} + d_i)}.$$

For the **MA model** the fixed point, where all alleles may have nonzero frequencies, is given by $(W_1^*, W_2^*, P_1^*, P_2^*)$, where for $i = 1, 2$,

$$W_i^* = \frac{u_i}{1 - c_{P_i}} \quad \text{and} \quad P_i^* = \frac{v_i}{1 - c_{P_i}}.$$

Besides the trivial solution of all alleles having frequency zero, the remaining solutions are given by $(W_1^*, 0, P_1^*, 0)$ and $(0, W_2^*, 0, P_2^*)$.

For the **iMA model** the fixed point, where all alleles may have nonzero frequencies, is given by $(W_1^{**}, W_2^{**}, P_1^{**}, P_2^{**})$, where for $i = 1, 2$,

$$W_i^{**} = \frac{u_i}{1 - c_{P_{3-i}}} \quad \text{and} \quad P_{3-i}^{**} = \frac{v_i}{1 - c_{P_{3-i}}}.$$

Besides all alleles having frequency zero, the remaining solutions are given by $(W_1^{**}, 0, 0, P_2^{**})$ and $(0, W_2^{**}, P_1^{**}, 0)$.

For the **GFG model** the fixed point, where all alleles may have nonzero frequencies, can be evaluated but is of complicated form. So we only note the results for $\beta_{ij} = \beta_i$, $\delta_{ij} = \delta_i$ and $s_{ij} = s_i$ (*i.e.* all of these parameters are independent of the parasite genotype). The fixed point

with nonzero entries is given by $(W_1^{***}, W_2^{***}, P_1^{***}, P_2^{***})$, where

$$W_1^{***} = \frac{(c_{P_2} - c_{P_1})u_1}{(1-c_{P_2})(1-c_{P_1})}, \quad W_2^{***} = \frac{u_2}{1-c_{P_1}}, \quad P_1^{***} = \frac{v_2 - v_1}{1-c_{P_1}} \quad \text{and} \quad P_2^{***} = \frac{v_1}{1-c_{P_2}}.$$

Besides all alleles having frequency zero, further solutions are given by $(0, W_2^{*}, 0, P_2^{*})$, $(W_1^{**}, 0, 0, P_2^{**})$ and $(0, W_2^{**}, P_1^{**}, 0)$.

For $c_{H_1} = c_{H_2} = c_{P_1} = c_{P_2} = 0$, we also obtain

$$W_1^{***} = 0, \quad W_2^{***} = -\hat{I}_{22} + u_2, \quad P_1^{***} = -\hat{I}_{22} + v_2 \quad \text{and} \quad P_2^{***} = 0,$$

where $\hat{I}_{22}$ denotes the equilibrium solution of the infected host genotype. With the additional assumption of equivalent rates and costs among both host and parasite genotypes, so that particularly $u_1 = u_2 = u$ and $v_1 = v_2 = v$, we further have

$$W_1^{***} = u\left(1 - \frac{\beta}{\delta + d}\hat{H}_2\right), \; W_2^{***} = u\,\frac{\beta}{\delta+d}\hat{H}_2, \; P_1^{***} = v\left(1 - \frac{\beta}{\delta+d}\hat{H}_2\right) \; \text{and} \; P_2^{***} = v\,\frac{\beta}{\delta+d}\hat{H}_2,$$

where $\hat{H}_2$ denotes the equilibrium solution of the second healthy host genotype.

For the **iGFG** model, the nontrivial equilibrium solution is equivalent to the MA model with $(W_1^{*}, 0, P_1^{*}, 0)$.

SI 4. Summary of analytical results for site frequency spectra and related statistics

Assume a model, where a haploid population is evolving according to Wright-Fisher dynamics forwards in time and being of size N_{ref} at (and before) time zero. A sample of n sequences is taken. Neutral mutations occur at unlinked and previously monomorphic sites at rate $\theta = 2\,N_{\text{ref}}\,\mu$, μ being the mutation rate per genome per generation. Scale time in units of N_{ref} generations and let $N_{\text{ref}} \to \infty$ to reach the diffusion limit. Thereby, the relative population size $N(t)/N_{\text{ref}}$ converges to the strictly positive and piecewise continuous scaling function $\rho(t)$. The site frequency spectrum is the distribution of the number of times a mutant allele is observed in the sample among the polymorphic loci.

The absolute site-frequencies over time $f_{n,i}(t)$, $1 \le i \le n-1$, with a mutation-drift equilibrium at time zero can be simply obtained from Equation 33 in [15] as

$$f_{n,i}(t) = \frac{\theta}{i} \sum_{k=2}^{n} (-1)^k (2k-1)\ {}_3F_2(n-i+1, k, 1-k; n+1, 2; 1) \left[R(0) + \binom{k}{2} \int_0^t R(s) ds \right],$$

where ${}_3F_2(a,b,c;d,e;z) = \sum_{l \ge 0} (a_{(l)} b_{(l)} c_{(l)})/(d_{(l)} e_{(l)}) z^l/l!$, with $p_{(0)} = 1$ and $p_{(l)} = p(p+1)\cdots(p+l-1)$ for $l \ge 1$, is a *generalized hypergeometric function*, and $R(s) = \exp\left[-\binom{k}{2} \int_s^t \rho^{-1}(u) du\right]$.

The relative site frequencies over time $r_{n,i}(t)$ are obtained as $r_{n,i}(t) = f_{n,i}(t)/\sum_{k=1}^{n-1} f_{n,k}(t)$, where the denominator gives the absolute number of segregating sites, $S_n(t)$. For the average number of pairwise differences, we have $\Pi_n(t) = 1/\binom{n}{2} \sum_{k=1}^{n-1} k(n-k) f_{n,k}(t)$.

These implementations can be computationally demanding since the inverse of the relative population size function $\rho(t)$ has to be integrated numerically with high precision before being applied to an exponential function that has to be integrated numerically again from the initial time zero up to the time points at which the SFS are evaluated. These exponentials also cover binomial coefficients that depend on sample size and the numerical integration has to be performed for every single one of those. Therefore, an increasing amount of integration steps and computation time is required with increasing sample size, so that we only consider a rather small sample size of twenty individuals for which the SFS of a single time point can be rather quickly obtained and even for later time points. This is particularly desirable, since we are interested in taking samples recurrently over the course of time.

SI 5. Stability behavior of the fixed points for two alleles

SI 5.1. The matching-allele model

Table SI 5.1.1 Legend of coloring for possible allele frequency changes

color: type of allele frequency change	exemplary figure
black: loss of all alleles	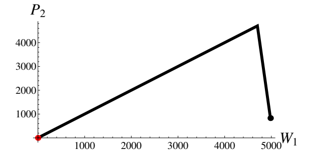
blue: H_1 and H_2 increase towards infinity;<br>loss of all infected alleles	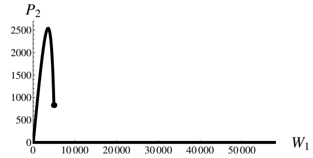
orange: limit cycle; I_{12} and I_{21} get lost	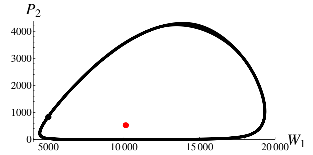
red: circular to rectilinear motion<br>towards a stable equilibrium;<br>I_{12} and I_{21} get lost;	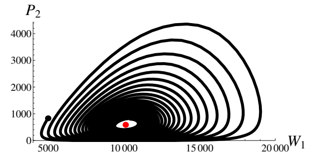
green: H_1 and H_2 converge to certain numbers;<br>I_{12} and I_{21} get lost;<br>I_{11} and I_{22} increase towards infinity	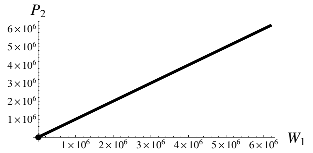

In the left column the possible fates of (healthy and infected) genotypes are summarized and assigned to colors. In the right column corresponding exemplary parametric plots of host and parasite allele frequencies are shown using Equations (1) and (2). The black dot depicts the initial allele frequency, whereas the red dot shows the zero point in the first and the fixed point in the third and in the fourth subfigure.

Figure SI 5.1.1

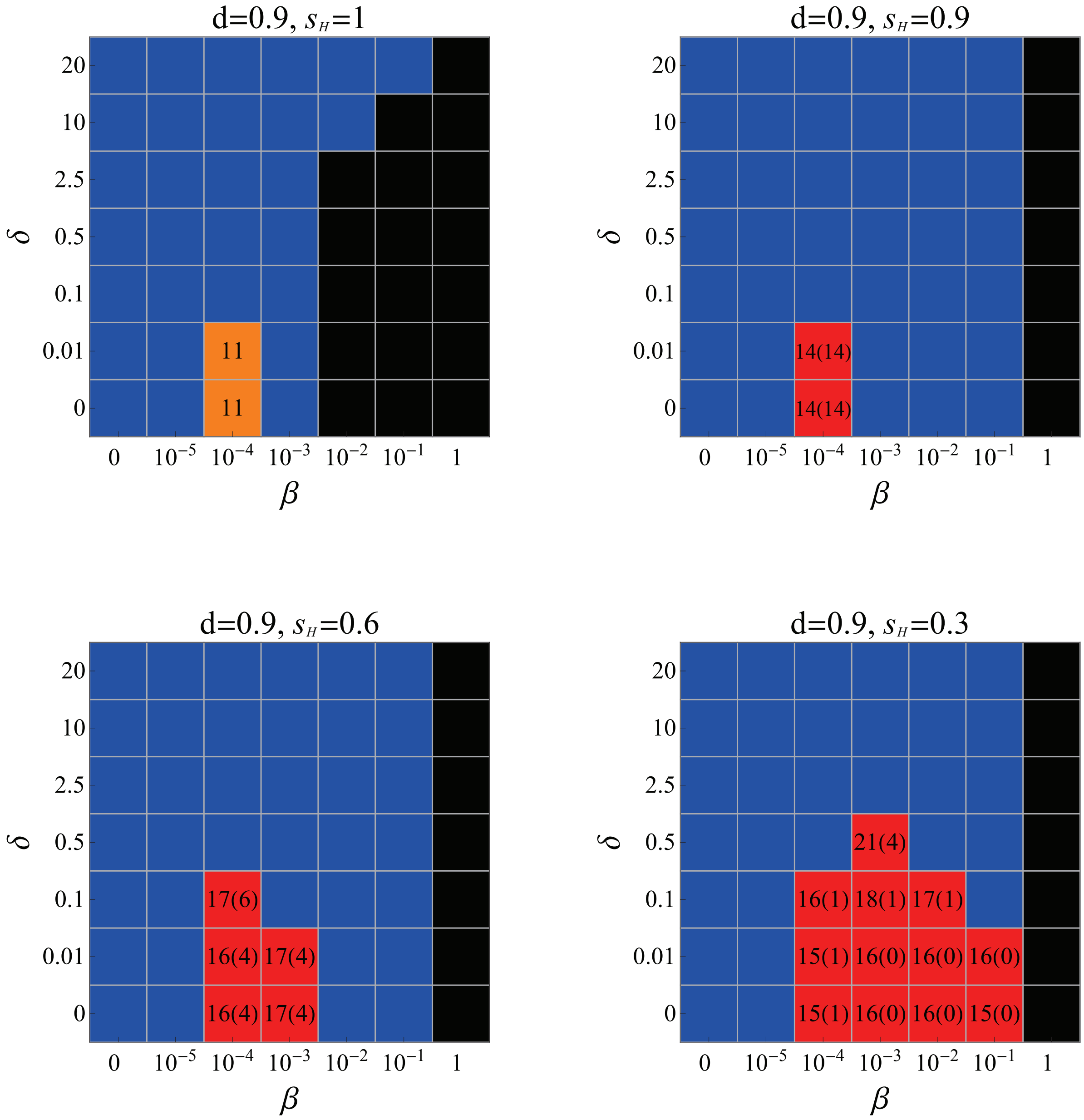

Figure SI 5.1.2

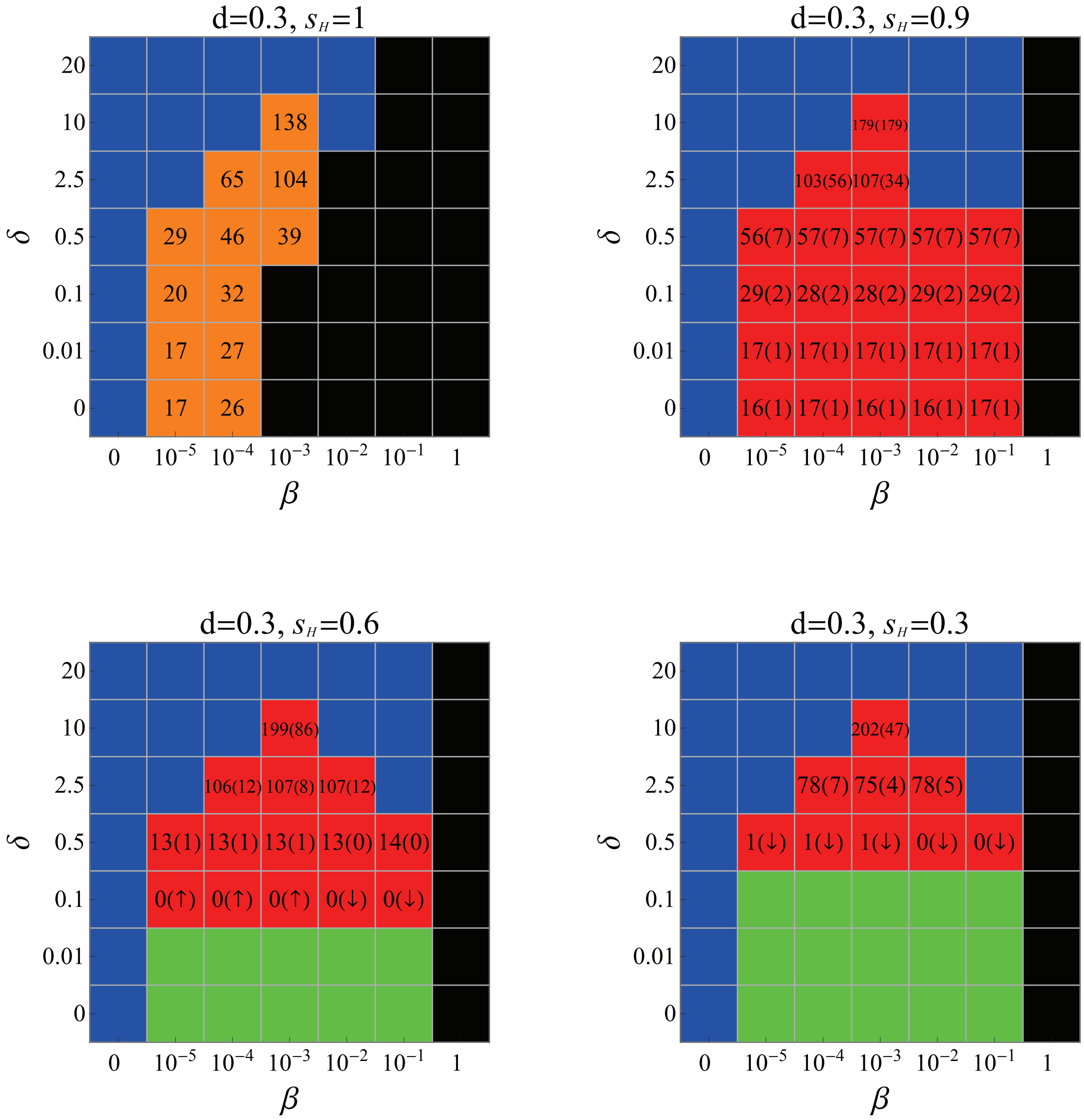

Description of the results

For each panel of Figures SI 5.1.1 and SI 5.1.2 the system of differential equations (1) and (2) were numerically evaluated for $b = 1$, $c_P = c_H = 0.05$ and the specified parameters over 500 time steps. The various possible coevolutionary scenarios of hosts and parasites are color-coded and summarized in detail in Table SI 5.1.1. The squares featuring numbers represent the parameter combinations for which cycling around or immediate attainment of the fixed point of N^{W} is obtained. For $s = 1$, the amplitude of the cycles around the fixed point of N^{W} remains constant over time. For other values of s we distinguish two cases: We first evaluated the total amount of cycles around the fixed point of N^{W} based on a numerical accuracy of 20 digits. Since the cycling dumps off in these cases, we also note in brackets the number of cycles until the amplitude is enclosed by the interval $[0.95\, N^{\mathrm{W}}, 1.05\, N^{\mathrm{W}}]$. The upwards and downwards arrows in some of the brackets illustrate expansions and declines to the fixed point, respectively.

SI 5.2. The gene-for-gene model

Table SI 5.2.1 Legend of coloring for possible allele frequency changes

color: type of allele frequency change	exemplary figure
green: H_1 and H_2 converge to certain numbers<br>(H_1 can get lost); I_{11} gets lost;<br>I_{21} and I_{22} increase towards infinity.<br>I_{12} increases towards infinity (dark green),<br>or gets lost (light green)	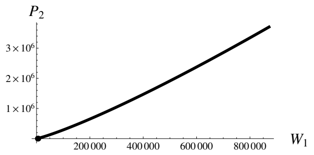
orange: limit cycle; H_1, I_{11} and I_{12} get lost.<br>I_{21} (dark orange) or<br>I_{22} (light orange) gets lost	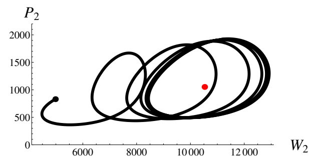
red: circular to rectilinear motion<br>towards a stable equilibrium;<br>H_1, I_{11} and I_{12} get lost.<br>I_{21} (dark red) or<br>I_{22} (light red) gets lost	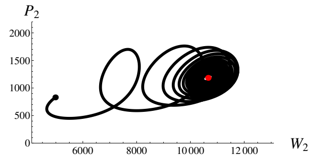
yellow: H_1 increases towards infinity;<br>loss of all the other alleles	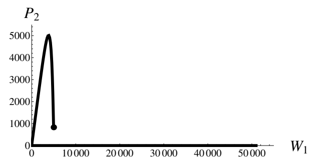
gray: H_2 converges to a certain number;<br>H_1, I_{11} and I_{12} get lost.<br>I_{22} increases towards infinity and<br>I_{21} gets lost (dark gray),<br>or vice versa (light gray)	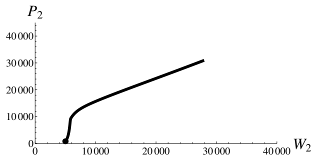

In the left column the possible fates of (healthy and infected) genotypes are summarized in addition or slightly modified to the scenarios already presented in Table SI 5.1.1 by means of the MA model. In the right column exemplary parametric plots of host and parasite allele frequencies are shown (in the first three panels for the dark color). The black dot depicts again the initial allele frequency.

Figure SI 5.2.1

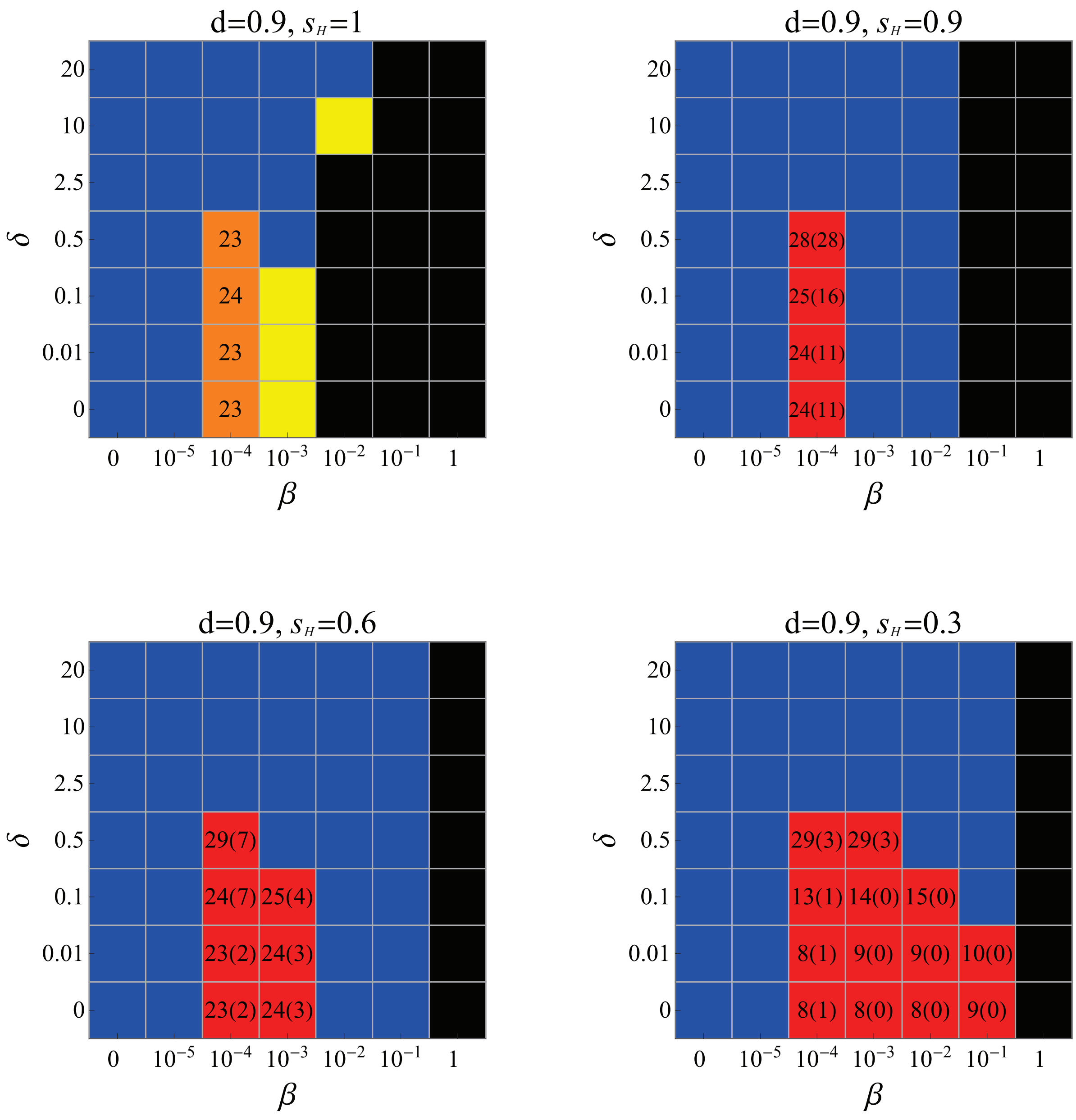

Figure SI 5.2.2

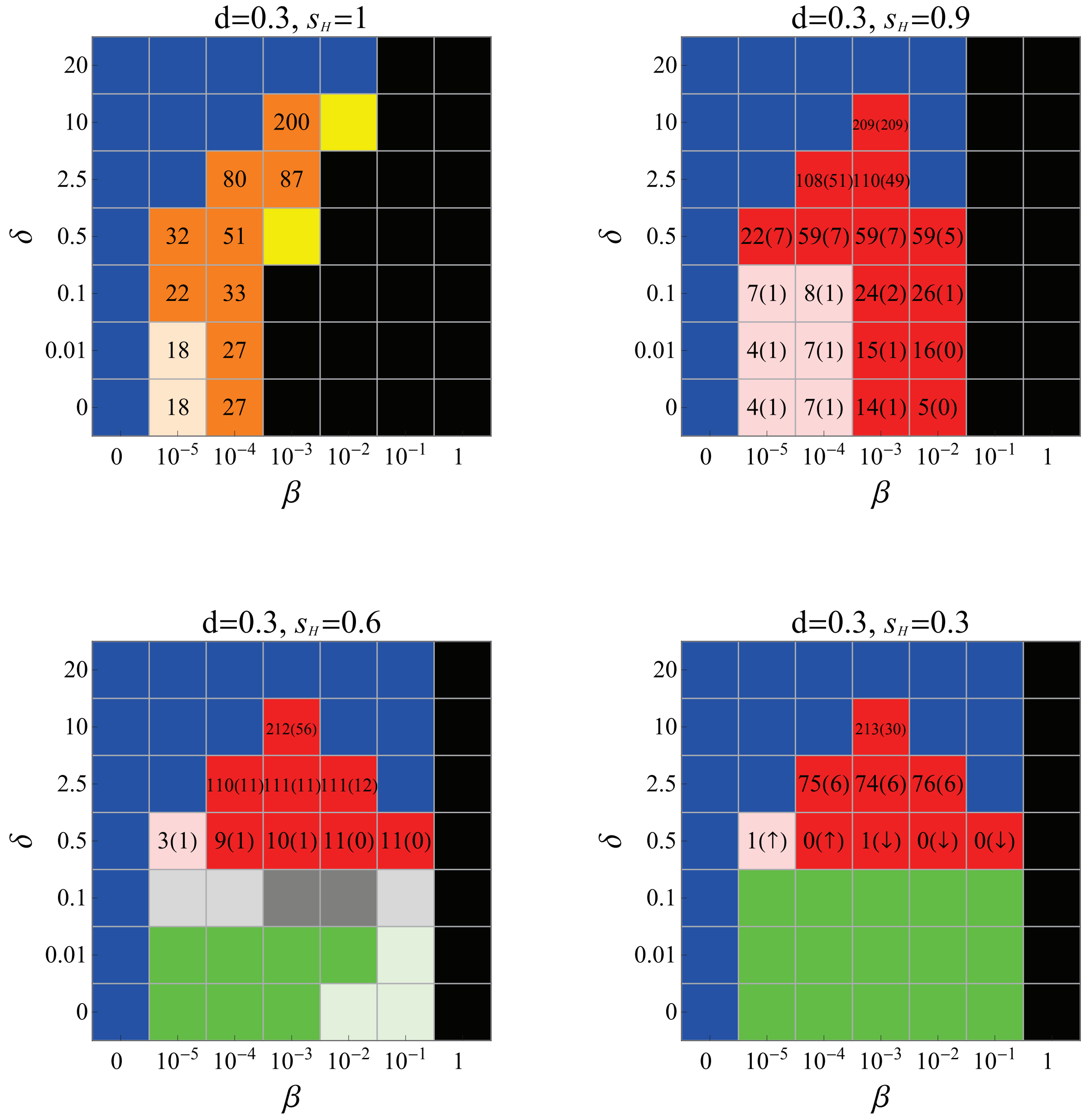

Description of the results

For each panel of Figures SI 5.2.1 and SI 5.2.2 the system of differential equations (1) and (2) were numerically evaluated for $b = 1$, $c_{H_1} = c_{P_2} = 0.05$, $c_{H_2} = c_{P_1} = 0$ and the specified parameters over 500 time steps. The various possible coevolutionary scenarios of hosts and parasites are color-coded and summarized in detail in Tables SI 5.1.1 and SI 5.2.1; the latter table summarizes cases either slightly different for or even exclusive to the GFG model due to the additional possibility of infection. The squares featuring numbers represent the parameter combinations for which cycling around or immediate attainment of the fixed point of N^{W} is obtained. For $s = 1$, the amplitude of the cycles around the fixed point of N^{W} remains constant over time. For other values of s we distinguish two cases: We first evaluated the total amount of cycles around the fixed point of N^{W} based on a numerical accuracy of 20 digits. Since the cycling dumps off in these cases, we also note in brackets the number of cycles until the amplitude is enclosed by the interval $[0.95\,N^{\mathrm{W}}, 1.05\,N^{\mathrm{W}}]$.

SI 6. Parametric plots for a matching-allele example

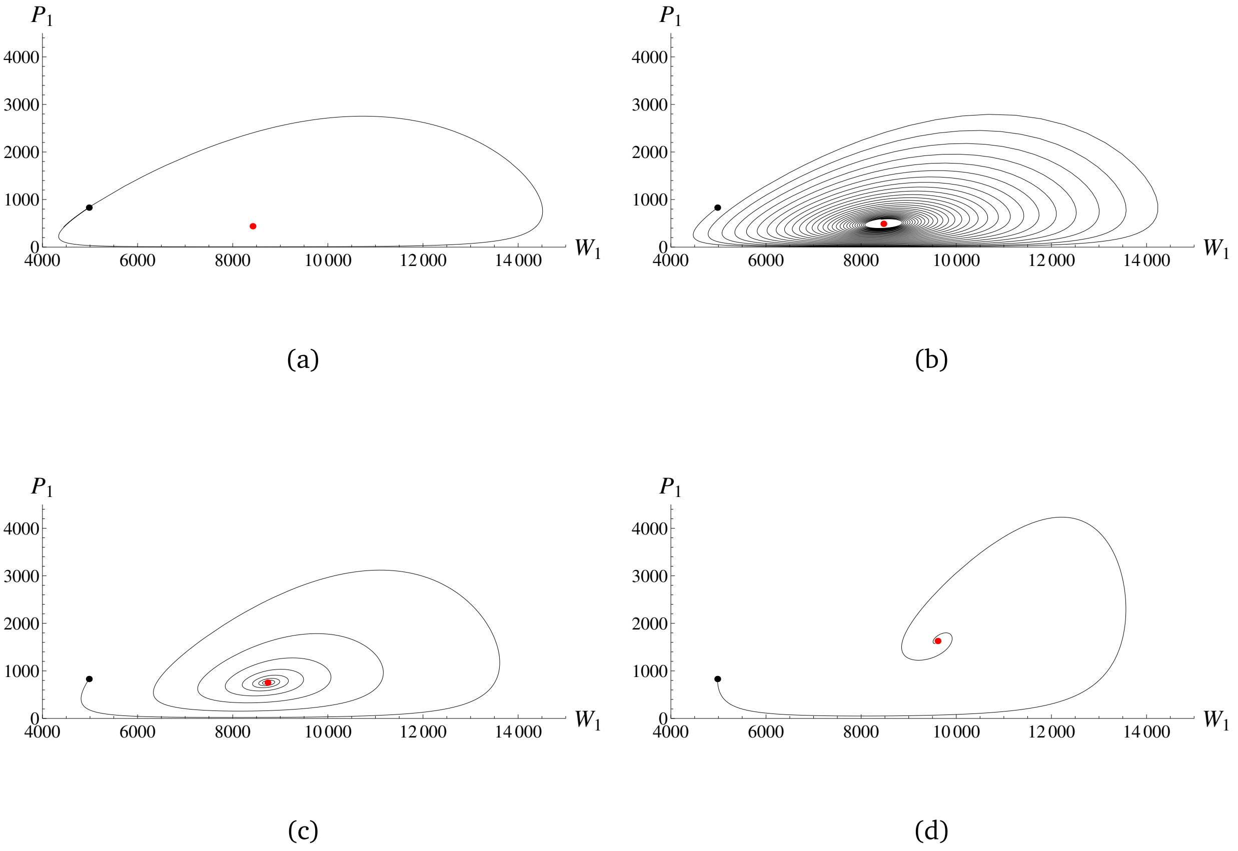

Figure SI 6.1 Parasite and host alleles of genotype one are plotted against each other for the MA model over time by numerically solving (1) and (2) for the following parameters (being equivalent for both genotypes): $b = 1$, $d = 0.9$, $\delta = 0.01$, $\beta = 0.00012$ and $c_P = c_H = 0.05$. The initial conditions are $H_1 = H_2 = 4150$ and $I_{11} = I_{12} = I_{21} = I_{22} = 415$. The selection coefficients s are given by (a) 1, (b) 0.9, (c) 0.6 and (d) 0.3. The parametric plots are shown for (a) 40, (b) 900, (c) 225 and (d) 76 time steps, which are the minimum amounts of time to complete one circle (a), come close to the fixed point (b), or to reach the fixed point (c), (d). The initial and fixed points are respectively colored in black and red.

SI 7. Time scaling and the SFS of three allelic classes over time for a matching-allele example

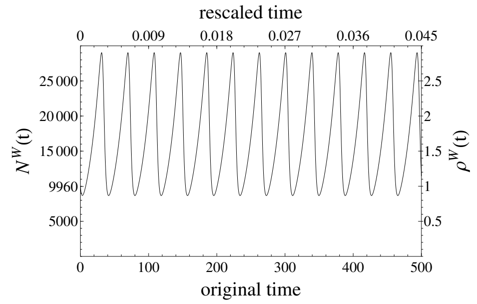

(a)

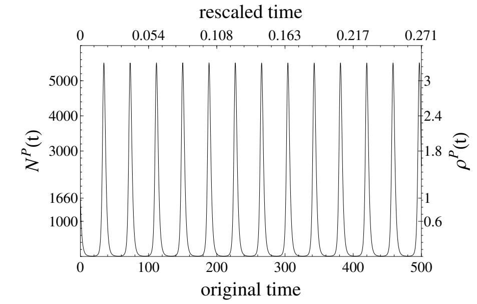

(b)

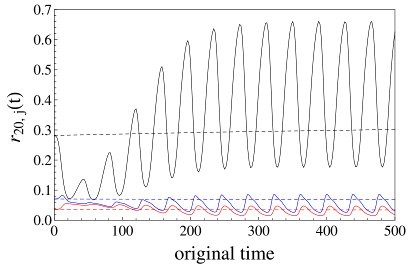

(c)

Figure SI 7.1 Population size changes in the host (a) and in the parasite (b) are generated for the MA model via the parameters $b = 1, d = 0.9, \delta = 0.01, \beta = 0.00012, c_P = c_H = 0.05$, and $s = 1$. The initial conditions are $H_1 = H_2 = 4150$ and $I_{11} = I_{12} = I_{21} = I_{22} = 415$, so that the reference population sizes N_{ref} of the host and the parasite are respectively given by 9960 and 1660 before their interaction starts at time zero. In both cases, the lower x-axes show time at the original scale of the dynamical system, whereas time is scaled by the respective values of $N_{\text{ref}} \cdot 1/d$ for the upper x-axes. The left y-axes denote the absolute values of the changing population size and the right y-axes denote the relative values as $\rho(t) = N(t)/N_{\text{ref}}$. The number of infected alleles fluctuate between about ten and 5.500 individuals. We evaluated the SFS for every second generation (on the original scale) in both cases and plot (c) $r_{20,j}(t)$ for $j = 1$ (black), $j = 3$ (blue) and $j = 8$ (red) against time for the host (dashed) and the parasite (solid). Note that the SFS shows similar results for all $j \geq 8$.

SI 8. Time scaling and the distribution of singletons over time for a gene-for-gene example

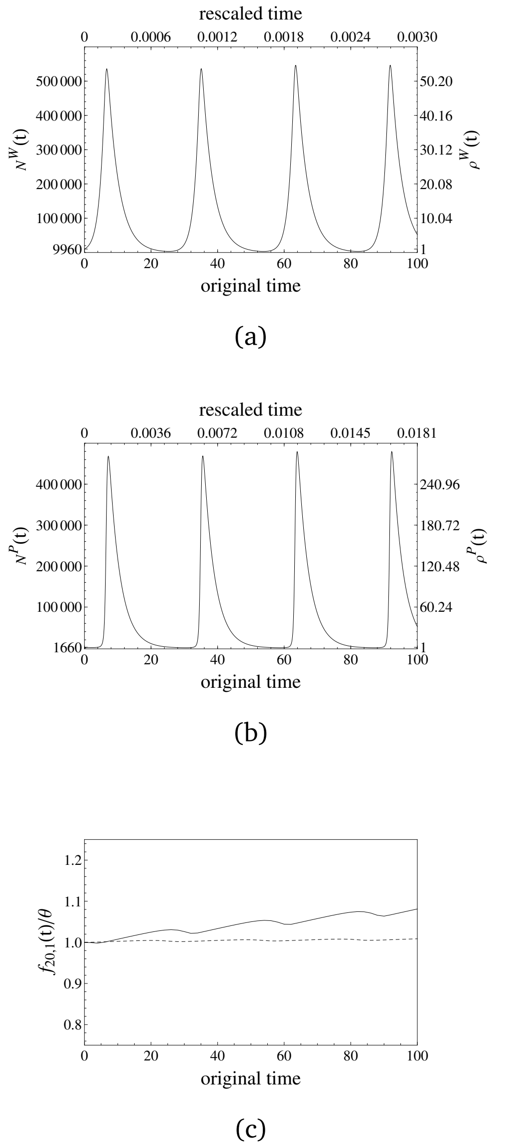

Figure SI 8.1 Population size changes in the host (a) and in the parasite (b) are generated for the GFG model via the parameters $b = 1, d = 0.3, \delta = 0, \beta = 0.00001, c_{H_1} = c_{P_2} = 0.05, c_{H_2} = c_{P_1} = 0$ and $s = 1$. The initial conditions are $H_1 = H_2 = 4150$ and $I_{11} = I_{12} = I_{21} = I_{22} = 415$, so that the reference population sizes N_{ref} of the host and the parasite are given by 9960 and 1660, respectively, before their interaction starts at time zero. In both cases, the lower x-axes show time at the original scale of the dynamical system, whereas time is scaled by the respective values of $N_{\text{ref}} \cdot 1/d$ for the upper x-axes. The left y-axes denote the absolute values of the changing population size and the right y-axes denote the relative values as $\rho(t) = N(t)/N_{\text{ref}}$. The number of infected alleles fluctuate between about 720 and 478.000 individuals. We evaluated allelic spectra for every second generation (on the original scale) and (c) plot the absolute number of singletons scaled by θ, $f_{20,1}(t)/\theta$, against time for host (dashed) and parasite (solid). Note that the SFS shows barely any changes over time for all the remaining allelic classes for host and parasite.

SI 9. Distributions of singletons over time for various initial conditions

SI 9.1. A gene-for-gene example for 2%, 20% and 50% initially infected alleles

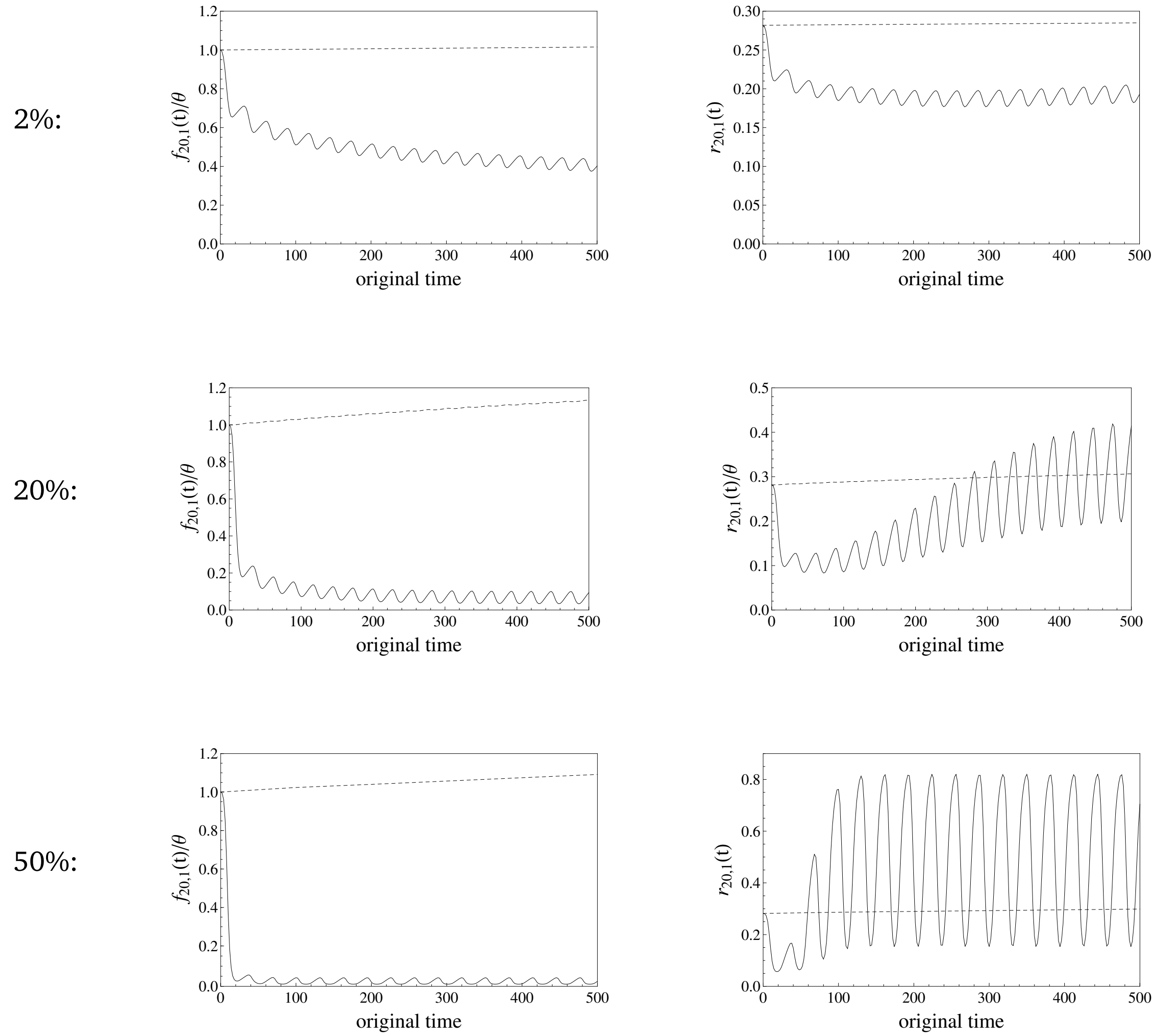

Figure SI 9.1 Population size changes in the host and in the parasite are generated for the GFG model via the parameters $b = 1, d = 0.9, \delta = 0.01, c_{H_1} = c_{P_2} = 0.05, c_{H_2} = c_{P_1} = 0, s = 1$ and $\beta = 0.000005$ (2%) or $\beta = 0.00005$ (20% and 50%). The initial conditions are $H_1 = H_2 = 49500$ and $I_{11} = I_{12} = I_{21} = I_{22} = 500$ (2%); $H_1 = H_2 = 4150$ and $I_{11} = I_{12} = I_{21} = I_{22} = 415$ (20%); $H_1 = H_2 = 3333$ and $I_{11} = I_{12} = I_{21} = I_{22} = 833$ (50%). Therefore, the reference population sizes N_{ref} of the host and the parasite are, respectively, given by 101000 and 2000 (2%); 9960 and 1660 (20%); 9998 and 3332 (50%) before their interaction starts at time zero. We evaluated allelic spectra for every second generation (on the original scale) in all cases and plot the absolute number of singletons scaled by θ, $f_{20,1}(t)/\theta$, (left panels) and the relative number of singletons $r_{20,1}(t)$ (right panels) against time for the host (dashed) and the parasite (solid).

SI 9.2. A matching-allele example for 2%, 20% and 50% initially infected alleles

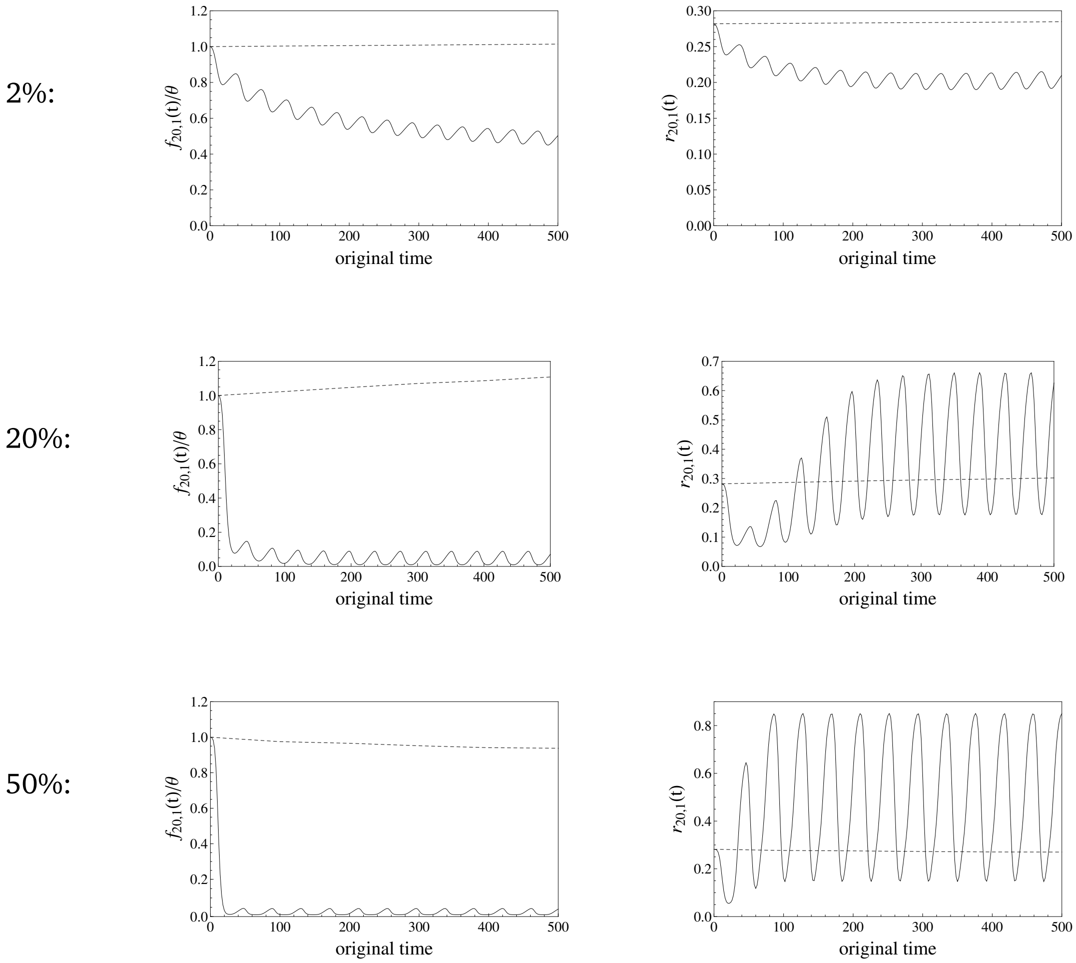

Figure SI 9.2 Population size changes in the host and in the parasite are generated for the MA model via the parameters $b = 1, d = 0.9, \delta = 0.01, c_H = c_P = 0.05, s = 1$ and $\beta = 0.0000012$ (2%), $\beta = 0.000012$ (20%) and $\beta = 0.00005$ (50%). The initial conditions are $H_1 = H_2 = 49500$ and $I_{11} = I_{12} = I_{21} = I_{22} = 500$ (2%); $H_1 = H_2 = 4150$ and $I_{11} = I_{12} = I_{21} = I_{22} = 415$ (20%); $H_1 = H_2 = 3333$ and $I_{11} = I_{12} = I_{21} = I_{22} = 833$ (50%). Therefore, the reference population sizes N_{ref} of the host and the parasite are, respectively, given by 101000 and 2000 (2%); 9960 and 1660 (20%); 9998 and 3332 (50%) before their interaction starts at time zero. We evaluated allelic spectra for every second generation (on the original scale) in all cases and plot the absolute number of singletons scaled by θ, $f_{20,1}(t)/\theta$, (left panels) and the relative number of singletons $r_{20,1}(t)$ (right panels) against time for the host (dashed) and the parasite (solid).

SI 10. The impact of multiple parasites per host and polycyclic diseases on detecting cycling population sizes for a gene-for-gene interaction

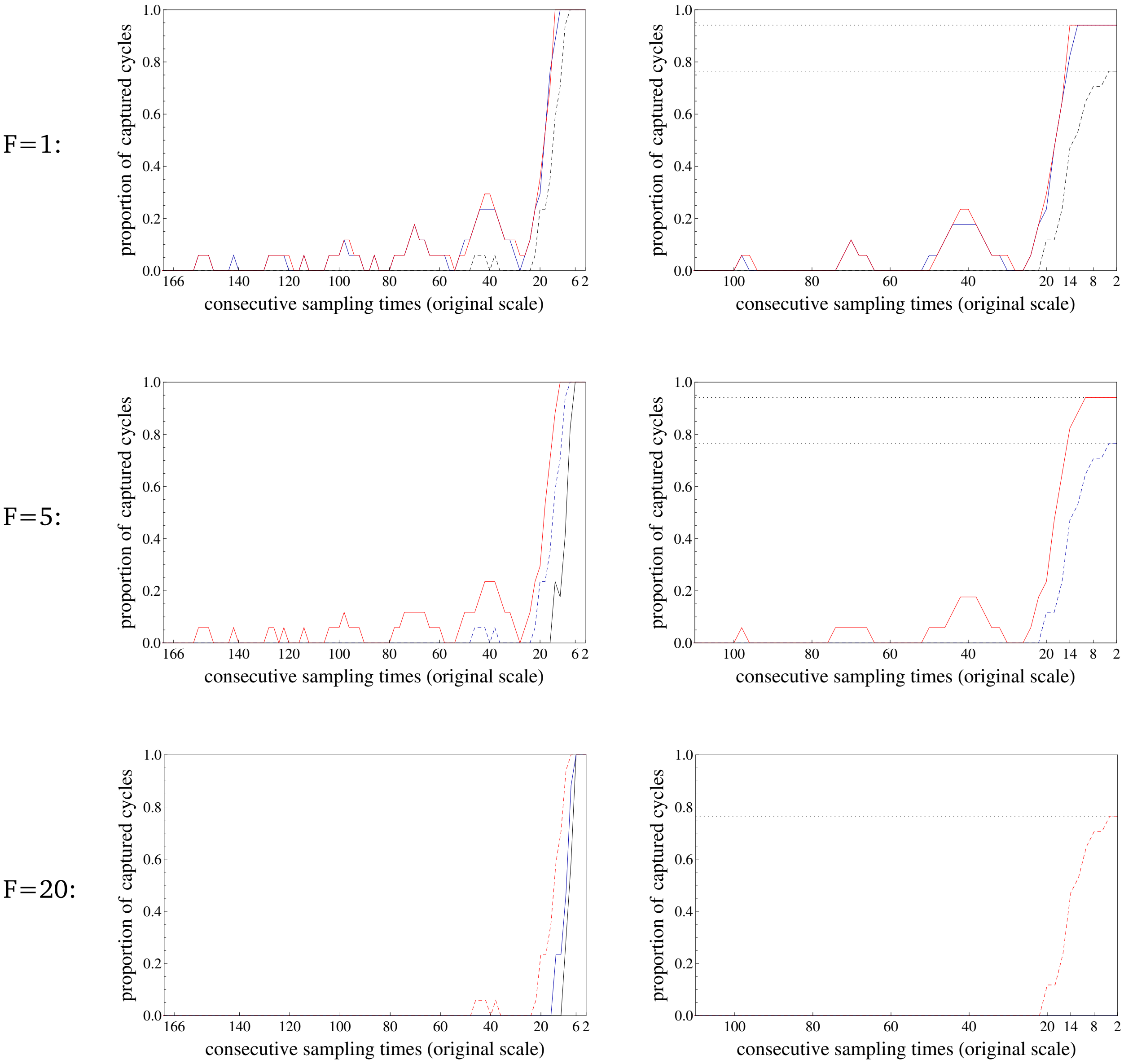

Figure SI 10.1

Description of the results

The dynamical system (1) and (2) was evaluated for the GFG model and the same parameter set as in the main example of *Results* (see Figure 3). Various values were considered for the number of parasite generations per host generation ($E = 1$, black curves; $E = 5$, blue curves; $E = 20$, red curves) and for the number of parasites per host (F) taking the same values as E. Trajectories of the population size changes of the parasite were obtained for these nine parameter combinations and respectively employed into the analytical equation of the SFS, which was evaluated at every second generation (on the original scale) over an interval of 500 time points. Based on these datasets, we evaluated $\Pi_{20}(j \cdot k/N_{\text{ref}})/\theta$ (see SI 4) for $j = 2, 4, 6 \ldots, 166$, and $k = 0, 1, 2, \ldots,$ up to $j \cdot k$ taking the largest value equal or smaller than 500. Four time points are at least required to capture a single cycle and the value $j = 166$, for instance, means that a sample is taken every 166*th* generation at times zero, 166, 332 and 498. For every j_0, the times of alternating local minima and maxima of $\Pi_{20}(j_0 \cdot k/N_{\text{ref}})$ were obtained over the values of k, before the rate of change as defined by roc=$(\Pi_{20}((m+1)/N_{\text{ref}}) - \Pi_{20}(m/N_{\text{ref}}))/\Pi_{20}(m/N_{\text{ref}})$, $m = 0, 1, 2, \ldots,$ was applied until the end of the interval is reached. In all plots, the number of captured cycles are plotted (relative to the total number of cycles of the time interval) against consecutive sampling times j_0. For the left-hand panels, all cycles are considered, whereas for the right-hand panels only cycles are considered, if the rate of change between two consecutive extrema deviates by at least two percent, *i.e.* roc≥ 0.02. Every cycle is captured when sampling at every sixth generation on the left-hand side whereas not all cycles can be captured in any case and not at all for all chosen values of $F < E$ on the right-hand side. The dashed lines illustrate cases with $E = F$ giving equivalent curves. Also note that the number of captured cycles does not increase monotonically with the number of sampling times until sampling at every 20th generation in all the examples because a smaller number of sampling points (*e.g.* sampling at every 42nd generation in the first panel) can be more suitably distributed among the peaks and valleys of the considered time interval than a greater choice (*e.g.* sampling at every 30th generation in the first panel).
